# Activation‐induced changes in platelet surface receptor expression and the contribution of the large‐platelet subpopulation to activation

**DOI:** 10.1002/rth2.12303

**Published:** 2020-01-27

**Authors:** Masaaki Moroi, Richard W. Farndale, Stephanie M. Jung

**Affiliations:** ^1^ Department of Biochemistry University of Cambridge Cambridge UK

**Keywords:** activation, GPIb, GPVI, IIbIIIa, large platelets, receptors, αIIbβ3

## Abstract

**Objective:**

Platelet surface receptors are also present subcellularly in organelle membranes and can be expressed on the surface upon platelet activation. However, some receptors were reported to be decreased after activation. We analyzed the mechanism of activation‐dependent expression for different receptors.

**Methods:**

Flow cytometry using platelet‐rich plasma or washed platelets was used to analyze receptor‐expression changes after platelet activation by glycoprotein (GP) VI–specific agonists, crosslinked collagen‐related peptide (CRP‐XL) and convulxin (Cvx), and thrombin. Platelets prelabeled with fluorescent antibody specific for a receptor were allowed to adhere on immobilized collagen or fibrinogen and post‐stained with antibody against the same receptor labeled with another fluorophore, allowing us to differentiate preexisting receptors from newly expressed receptors.

**Results:**

Surface expression of αIIbβ3 increased in CRP‐XL–, Cvx‐, or thrombin‐stimulated platelets, but GPIb decreased due to shedding and internalization. Both total and dimeric GPVI increased in thrombin‐induced platelets, but decreased in platelets stimulated by Cvx, as a result of internalization. The larger platelets showed a greater increase in surface receptor (α2β1, αIIbβ3, GPVI, GPIb) expression upon activation compared to the smaller ones. Pre‐ and postlabeling with antibody specific for the same receptor, but conjugated with different fluorophores, allowed us to differentiate the receptors expressed on the surface of resting platelets from receptors newly exposed to the surface upon platelet activation.

**Conclusions:**

Increased receptor expressions after activation are mainly manifested in the larger platelets. On platelets adhered on fibrinogen, the newly expressed receptors, especially GPVI, are localized in the lamellipodia of the spread platelets.

**Essentials**

- Activation‐dependent platelet surface expression of different receptors was analyzed.
- Changes in surface expression depended on both the receptor and the platelet agonist.
- Newly expressed receptors localize on lamellipodia of platelets spread on fibrinogen.
- Increased receptor expressions upon activation are mainly manifested in larger platelets.

## 1 INTRODUCTION

Platelets are anucleate small blood cells, but they have several intracellular organelles and membrane systems whose localization and morphology are changed upon platelet activation by various stimulants. Activation transforms the smooth disclike shape of resting platelets to a disturbed spherical shape with numerous filopodial extrusions and lamellipodia, accompanied by marked changes in subcellular organelle localization. Secretory dense granules and α‐granules extrude their contents to the extracellular medium or through the inside space of the open canalicular system (OCS), and granule membranes fuse with the plasma or OCS membrane.^1^, ^2^, ^3^, ^4^ Major receptor proteins contained in the OCS and α‐granule membranes, including glycoprotein (GP) Ib and αIIbβ3,^1^, ^2^ become exposed to the surface when their membranes fuse with the platelet plasma membrane. This may explain increased surface αIIbβ3 expression in activated platelets,^1^, ^2^ but a decrease in surface GPIb^5^, ^6^ upon activation suggests that other mechanisms may be involved.

Platelets are essential for primary hemostasis since they adhere to subendothelial collagen exposed by vessel injury, become activated, aggregate, and form a thrombus to arrest bleeding. Hyperactive platelets, however, lead to formation of unwanted thrombi, which can detach and travel to distal areas, causing ischemic stroke or cardiovascular disease (CVD). Larger platelet size, measured as increased mean platelet volume (MPV), is a risk factor for cardiovascular disease CVD.^7^, ^8^ MPV increases with age in mice, which might explain the increasing CVD risk in the elderly.^9^ Circumstantial evidence suggests that large platelets are more active, but there is yet no direct evidence for this and why this may be so.

The aim of the present study is to compare larger platelets with the whole platelet population in terms of their surface expressions of receptors involved in thrombus formation in response to platelet activation using a clinically available method, flow cytometry. In resting platelets, surface expressions of GPIb, αIIbβ3, α2β1, and GPVI were higher in the larger platelets, commensurate with their larger surface area. Expressions of αIIbβ3 and α2β1 were increased in activated platelets, but GPIb and GPVI decreased due to shedding, internalization, or both. Increased exposure of intracellular receptors upon activation was most prominent in the larger platelets. These results suggest that platelets are a heterogeneous population, not only with respect to size but importantly with respect to activity and that the large platelets are the main determinants of platelet activation and function.

## 2 MATERIALS AND METHODS

### 2.1 Materials

GPVI dimer–specific, noninhibitory 204‐11 Fab^10^ was previously reported. Other mouse monoclonal antibodies: 1G5^11^ (anti‐pan GPVI; Biocytex, Marseille, France), anti‐GPIb antibodies AK2 (Novus Biologicals, Littleton, CO, USA) and clone 486805 (R&D Systems, Minneapolis, MN, USA); anti‐αIIbβ3 (M148; Abcam, Cambridge, UK); anti‐α2 (Gi9; Abcam), anti‐CD62P (AK6; Abcam); fluorescein isothiocyanate–conjugated antiactivated integrin αIIbβ3 procaspase‐activating compound‐1 (PAC‐1; BD Biosciences, San Jose, CA, USA). For some experiments, antibodies were labeled with Alexa Fluor‐488 or ‐647 by an Invitrogen labeling kit (Thermo Fisher Scientific, Waltham, MA, USA). Convulxin (Cvx)^12^ and crosslinked collagen‐related peptide (CRP‐XL)^13^ were previously reported.

### 2.2 Flow cytometry to measure surface receptor expression

Platelet‐rich plasma (PRP), prepared from acid‐citrate‐dextrose–anticoagulated blood from healthy volunteers,^10^ was diluted 4‐fold with modified HEPES (4‐[2‐hydroxyethyl]‐1piperazineethanesulfonic acid)–Tyrode’s buffer (HT: 136 mmol/L NaCl, 2.7 mmol/L KCl, 0.42 mmol/L NaH_2_PO_4_, 5.5 mmol/L glucose, 5 mmol/L HEPES, pH 7.4) or washed platelets (5 × 10^7^ cells/mL, HT) were prepared as before.^10^ Platelets were activated by CRP‐XL (5 μg/mL), Cvx (0.5 μg/mL), or thrombin (0.2 U/mL) for 4 minutes at 37°C. Samples were prepared for flow cytometry (Accuri C6, BD Biosciences, San Jose, CA, USA),^10^ with receptor‐specific primary antibody and Alexa Fluor‐488–conjugated anti‐mouse Fab (50 μg/mL; Jackson Immuno‐Research Laboratories, West Grove, PA, USA) as secondary antibody. The negative control was isotype control IgG or control mouse Fab. Platelet receptor expression was expressed as median fluorescence intensity (MFI) obtained for binding of receptor‐specific antibody.

**Figure 1:**
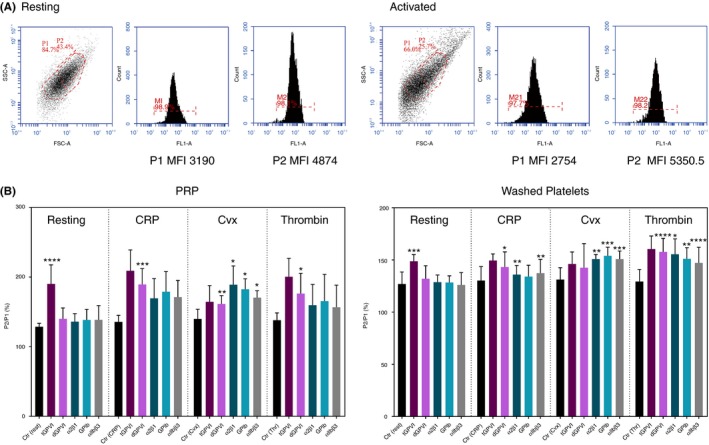
Flow cytometric analysis of large platelets. A, Resting and crosslinked collagen‐related peptide (CRP‐XL; 5 μg/mL)‐activated platelets in platelet‐rich plasma (PRP) were analyzed by flow cytometry. Both the resting and CRP‐XL–activated platelets were gated into 2 populations from their FSC/SSC plots (left plots): P1 (the total platelet population) and the larger half of P1 (P2). Since the Acuri C6 flow cytometer cannot delineate the larger half of the population from the lower half, we manually drew the gating line for each preparation analyzed. The fluorescence of P1 and that of P2, expressed as median fluorescence intensity (MFI), are shown in the middle and right plots for the resting and CRP‐activated platelets. B, Each P2/P1 ratio (reported as %) is calculated from the MFI of P1 and the MFI of P2. This figure summarizes from results for PRP and washed platelets, with different activators and specific antibodies (shown in parentheses) to determine expressions of total GPVI (1G5), GPVI‐dimer (204‐11 Fab), integrin α2β1 (Gi9), integrin αIIbβ3 (M148), and GPIb (AK2). Each bar in the graphs and their associated error bar show the mean ± standard deviation of the indicated number of determinations. P1/P2 for each receptor of platelets activated with CRP‐XL, convulxin (Cvx), or thrombin was compared to the respective P1/P2 of resting platelets. For PRP, CRP‐XL increased the ratio of GPVI‐dimer (*P* = .000, n = 5) and ratios of α2β1 (*P* = .08, n = 4) and αIIbβ3 (*P* = .08, n = 5) tended to be higher. Cvx also increased the ratio of GPVI‐dimer (*P* = .007, n = 6), α2β1 (*P* = .03, n = 4), GPIb (*P* = .02, n = 4), and αIIbβ3 (*P* = .02, n = 5). However, thrombin only increased the ratio of GPVI‐dimer (*P* = .03, n = 4). For washed platelets, the P2/P1 ratios are increased compared to those of the corresponding resting platelets after the activation by CRP‐XL (GPVI dimer: *P* = .01, n = 8; α2β1: *P* = .003, n = 5; GPIb: *P* = .11, n = 7; αIIbβ3: *P* = .002, n = 7); Cvx (GPVI dimer: *P* = .05, n = 7; α2β1: *P* = .002, n = 5; GPIb: *P* = .00, n = 7; αIIbβ3: *P* = .00, n = 6); and thrombin (GPVI‐dimer: *P*=<0.00, n = 13; α2β1: *P* = .02, n = 5; GPIb: *P* = .005, n = 7; αIIbβ3: *P* < .0001, n = 11). *P ≤ .05, **P ≤ .005, ***P ≤ .0005, ****P < .0001

In each experiment, the whole platelet population (P1) of a donor’s platelets was divided into 2 subpopulations according to size by gating in the forward scatter/side scatter (FSC/SSC) plot: The larger half was defined as P2 (larger platelets) and the smaller half was defined as P3 (smaller platelets; Figure 1A shows an example). Numbers of platelets in each subpopulation are calculated as percentage of total events.

### 2.3 Analysis of receptor expression in fixed platelets

Washed platelets (200 μL, 5 × 10^8^ cells/mL,) were activated (CRP‐XL or thrombin) for 4 minutes and mixed with 200 μL cold 8% formaldehyde/0.4% glutaraldehyde/PBS; after 30 minutes on ice, the mixture was diluted with 4 mL cold citrate‐buffered saline (CBS: 6.85 mmol/L citrate, 130 mmol/L NaCl, 4 mmol/L KCl, 5.5 mmol/L glucose buffer, pH 6.5) containing 5 mmol/L ethylenediaminetetraacetic acid (EDTA) and centrifuged in a microfuge (2100 rpm, 10 minutes). Each pellet was resuspended in 100 μL HT, and antibody binding was determined by flow cytometry. Formaldehyde/glutaraldehyde solution was used, as it has less effect on cell membrane phospholipid distribution^14^ and better maintained platelet antibody binding.

### 2.4 Immunoblotting

Washed platelets (200 μL, 10^9^ cells/mL) were activated with CRP‐XL (5 μg/mL), Cvx (0.5 μg/mL), or thrombin (0.5 U/mL) for 5 minutes at 37°C followed by 30 minutes at room temperature. Suspensions were centrifuged to separate supernatants and platelet pellets, which were dissolved with 200 μL 6 mol/L urea/1% SDS. Non‐reducing Laemmli's SDS sample buffer was added to each sample and heated at 100°C for 1 minute. Each sample (5 μL for the pellet, 15 μL for the supernatant) was electrophoresed on a 3% to 12% acrylamide SDS gel, immunoblotted with 1G5 or clone 486805 and analyzed by the Odyssey CLx (LI‐COR Biosciences, Lincoln, NE, USA).

### 2.5 Confocal imaging of internalization and surface receptor expression

#### 2.5.1 GPIb and GPVI internalization

Washed platelets were incubated with Alexa Fluor‐488–labeled 204‐11 Fab or clone 486805 mAb (4 μg/mL, 30 minutes) and washed once with CBS. The prelabeled platelets were activated with Cvx (0.5 μg/mL) or thrombin (0.5 U/mL) for 5 minutes at 37°C; added with an equal volume of ice‐cold 8% formaldehyde/0.4% glutaraldehyde/phosphate buffered saline (PBS), followed by 30‐minute on ice; washed once with 5 mmol/L EDTA in CBS; and resuspended in HT. Platelet suspensions were aliquoted on poly‐l‐lysine–coated MatTek dishes and centrifuged (2000 rpm, 5 minutes) in a Mistral 2000. Dishes were washed with PBS, blocked with 0.5% bovine serum albumin (BSA)/PBS, reacted with Alexa Fluor‐647–labeled M148 (anti‐CD41 mAb) for 60 minutes, and processed for imaging with an FV300 IX81 laser‐scanning confocal microscope with a 60 oil immersion objective (Olympus UK).^15^

#### 2.5.2 Receptors newly exposed upon platelet activation

Washed platelets prelabeled with Alexa Fluor‐488–labeled clone 486805 mAb, 204‐11 Fab or M148 were allowed to adhere to Horm‐collagen– or fibrinogen‐coated MatTek dishes (60 minutes, 37°C). Dishes were washed with PBS, and adhered platelets were fixed with 10% formaldehyde and blocked with 1% BSA/PBS. The platelets prelabeled with 204‐11 Fab, clone 48605 Mab, and M148 were reacted with Alexa Fluor‐647–labeled 1G5, 486805 Mab, and M148, respectively; washed; and imaged.

#### 2.5.3 Verification of GPVI internalization

1G5 was labeled with sulfo‐NHS‐SS‐biotin (Thermo Fisher Scientific, Waltham, MA, USA) to form the biotinylated antibody (1G5‐SS‐biotin). Washed platelets were incubated with 1G5‐SS‐biotin and Alexa Fluor 647–streptavidin. Then excess biotin was added; 10 minutes later, the mixture was diluted with CBS and centrifuged to obtain the platelet pellet. The pellet was resuspended in HT, and aliquots were activated with Cvx (1 μg/mL) or thrombin (0.2 units/mL), followed by addition of TCEP (tris[2‐carboxyethyl]phosphine); 28 mmol/L, final; Thermo Fisher Scientific, Waltham, MA, USA). Samples were processed for confocal microscopy to visualize the remaining GPVI‐associated fluorescence.

### 2.6 Statistical analysis

Paired Student’s *t*‐test (Prism v8.9, GraphPad Software, La Jolla, CA, USA) was applied to compare differences between activated platelets and their corresponding resting platelets.

## 3 RESULTS

### 3.1 Large platelets have higher levels of surface receptor expression

From the SSC/FSC scatter plot, resting platelets of each donor were gated into the larger platelets (P2) of the total population (P1; Figure 1A, resting platelets). Because each donor’s platelets showed a different size distribution, gating was tailored to each donor’s FSC/SSC plot. P2 of resting platelets showed 1.5‐fold higher binding of anti‐GPVI dimer (204‐11 Fab) than P1. CRP‐XL activation slightly changed the scatter plot (Figure 1A, activated) and P2, defined by the same gating as used for resting platelets, exhibited 1.9‐fold higher 204‐11 binding than P1.

Thus, we determined if the P2 population also showed higher numbers of other platelet surface receptors, using specific primary antibodies: total GPVI (1G5), GPIb (AK2), αIIbβ3 (M148), and α2β1 (Gi9), using isotype IgG (control) to determine background fluorescence (black bar [control] in Figure 1B) under each condition (resting, CRP, Cvx, thrombin).

P2/P1 of control resting platelets was about 130%, showing that P2 bound more control IgG than P1, indicating that P2 platelets have a larger cell surface area. For all receptors, except for total GPVI, P2/P1 ratios were similar to that of the control. The higher P2/P1 for total GPVI (Figure 1B: PRP, *P* < .0001, n = 7; washed platelets, *P* < .0004, n = 5) is due to the ability of 1G5 to activate platelets.^11^

For PRP added with agonist, only some of the P2/P1 data were significantly higher than the respective resting control: for CRP: dGPVI (*P* < .0001, n = 5); for Cvx: dGPVI (*P* < .007, n = 5), α2β1 (*P* < .028, n = 4), GPIb (*P* < .020, n = 4), αIIbβ3 (*P* < .019, n = 5); for thrombin: dGPVI (*P* < .025, n = 4). Although the P2/P1 for each receptor was higher than the ratio in the resting platelets for each donor, there was a high variability among values for different donors. The high fibrinogen level in PRP may have had some effect on antibody binding in the activated platelets, so we measured P2/P1 in washed platelets for most of our other experiments.

In contrast to PRP, activated washed platelets show higher P2/P1 for the examined receptors, except for dGPVI and GPIb in Cvx‐ and CRP‐induced platelets, respectively. Platelets are a heterogeneous population with respect to surface receptor expression in both the resting state and the activated state. The washed‐platelet results suggest that among the activated platelets, the larger platelet population showed the highest increase in surface receptor expression.

### 3.2 Receptor expression after platelet activation

**Figure 2:**
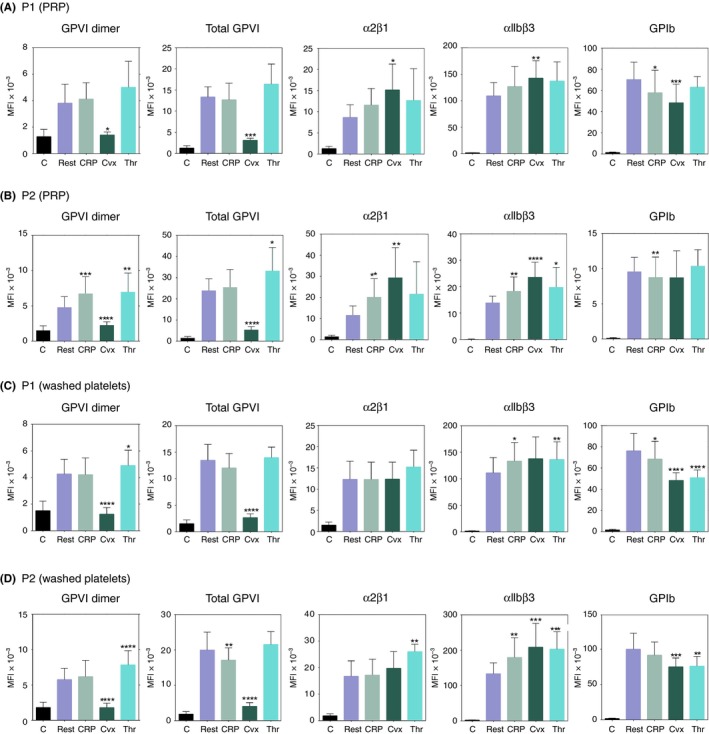
Changes of receptor expression after platelet activation. Receptor expressions are measured by flow cytometry using different agonists and receptor‐specific antibodies. Receptor expressions in platelets activated by CRP, convulxin (Cvx), or thrombin (Thr) are compared to the respective value in resting platelets (rest). A, P1 (total platelet population) of platelet‐rich plasma (PRP); B, P2 subpopulation of PRP (A); C, P1 of washed platelets; D, P2 subpopulation of washed platelets (C). Each bar in the graphs and their associated error bar show the mean ± SD of the indicated number of determinations. *P ≤ .05, **P ≤ .005, ***P ≤ .0005, ****P < .0001. Detailed descriptions of the statistical analyses are given in the Results section

Surface receptor expressions in platelets activated by CRP‐XL, Cvx, and thrombin were determined and compared to expression of each receptor in resting platelets (Figure 2). For the total platelet population (P1) of PRP (Figure 2A), both total GPVI and GPVI dimer very markedly decreased after stimulation by Cvx (GPVI dimer: *P* = .03, n = 5; total GPVI: *P* = .000, n = 4), but no significant changes in expression of either in response to CRP‐XL or thrombin. α2β1 and αIIbβ3 expressions tended to increase after activation but not significantly, and only Cvx induced significant increases (α2β1: *P* = .02, n = 4 and αIIbβ3: *P* = .004, n = 5). Each agonist decreased surface GPIb expression: CRP‐XL (*P* = .02, n = 5), Cvx (*P* < .005, n = 5), and thrombin (*P* = .08, n = 5).

P1 of washed platelets (Figure 2C) showed changes similar to those for P1 of PRP, but they became more significant because of the larger number of measurements. GPVI expression is strongly decreased by Cvx (GPVI dimer: *P* < .0001, n = 7; total GPVI: *P* < .0001, n = 6). GPVI dimer, but not total GPVI, is slightly increased by thrombin (*P* = .0158, n = 13). None of the agonists changed α2β1 expression. αIIbβ3 expression tended to increase after activation (CRP‐XL: *P* = .03, n = 7; Cvx: *P* = .08, n = 6; thrombin: *P* = .003, n = 11). Surface GPIb expression, however, strongly decreased in response to each agonist: CRP‐XL (*P* = .02, n = 7), Cvx (*P* < .0001, n = 7), and thrombin (*P* < .0001, n = 7).

If we compare the differences in receptor expression in the P2 population of PRP and washed platelets (Figure 2B,D), which has higher expression of each receptor than the total population (P1), the changes in surface receptor expression upon activation are even more dramatic.

**Figure 3:**
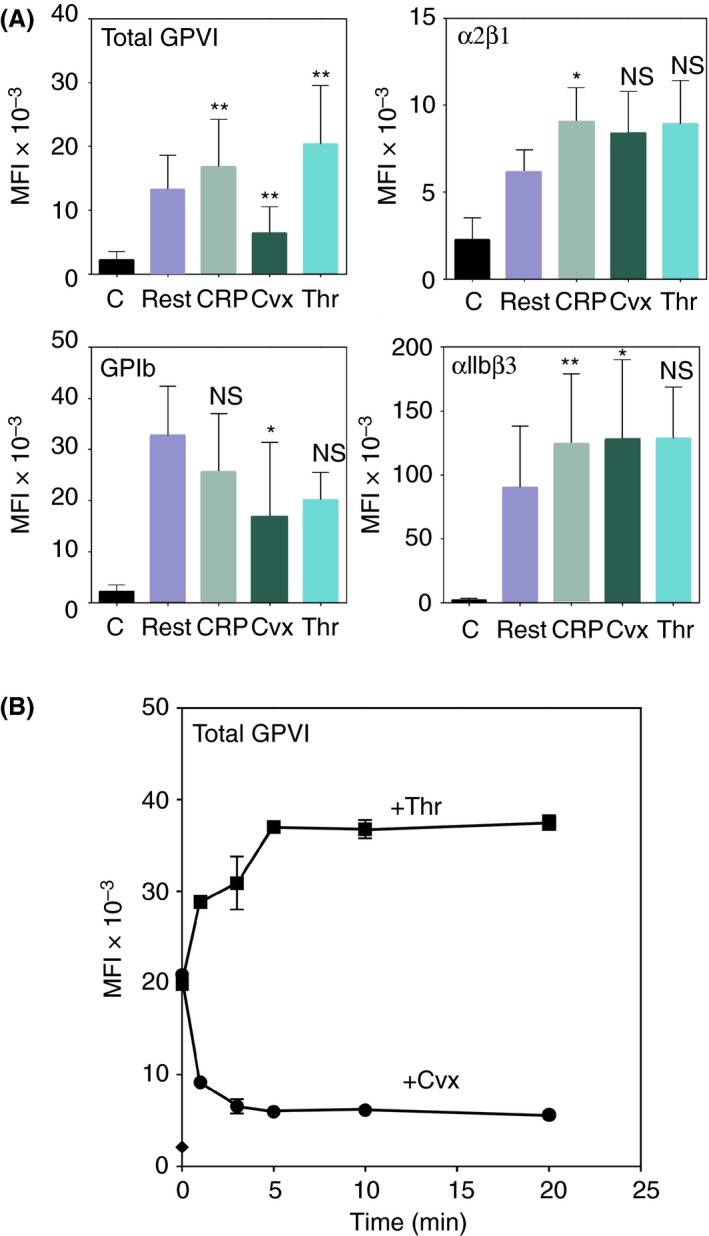
Analyses of receptor expressions using fixed platelets. A, Receptor expressions in fixed platelets. After platelets were activated, as described in Figure 2, they were fixed, stained using specific antibodies for the indicated receptors, and receptor expressions determined by flow cytometry. The increase or decrease of specific receptors in fixed platelets were similar to those obtained with native platelets. GPVI expression is decreased by convulxin (Cvx; *P* = .003, n = 8), but increased by crosslinked collagen‐related peptide (CRP‐XL) and thrombin (*P* = .003 and *P* = .003, respectively, n = 8). Integrin α2β1 tended to increase, although not reaching statistical significance, in platelets activated by CRP‐XL (*P* = .03, n = 4), Cvx (*P* = .13, n = 3), or thrombin (*P* = .06, n = 3). αIIbβ3 increased after the activation by CRP‐XL (*P* = .002, n = 4) or Cvx (*P* = .03, n = 3) and tended to increase in platelets activated by thrombin (*P* = .12, n = 3). The expression of GPIb decreased significantly in platelets activated by Cvx (*P* = .02, n = 3) and tended decrease in platelets activated by CRP‐XL (*P* = .19, n = 4) or thrombin (*P* = .07, n = 3). The coulumms show the means of the measurements and the error bars shows their SD. *P ≤ .05, **P ≤ .005, ***P ≤ .0005, ****P < .0001. B, Time course of the change in GPVI expression after activation. Washed platelets were activated by thrombin (0.5 units/mL) or Cvx (1 μg/mL) and incubated at 37°C. After the indicated time, aliquots of platelets were removed, fixed, and then analyzed by flow cytometry for total GPVI using 1G5 antibody, as described in the Methods section. Expression of total GPVI is increased by thrombin and decreased by Cvx in a rapid time course

To confirm these results, we determined receptor expression in stimulated washed platelets that were subsequently fixed with formaldehyde/glutaraldehyde; 204‐11 Fab was not used in these experiments, as it does not react with fixed platelets. These activated and fixed platelets showed similar receptor changes; Cvx, especially, strongly reduced GPVI expression (Figure 3A).

### 3.3 Time course and agonist‐concentration dependency of receptor expression in activated platelets

Flow cytometry of activated platelets (Figure 3B) showed that total GPVI expression is decreased by Cvx but increased by thrombin in a similar time scale: changes were evident by 0.5 minutes in some experiments, well detectable by 1 minute, and maximal before 5 minutes. Thus, the receptor‐expression changes are rapid, on the time scale of platelet aggregation.

**Figure 4:**
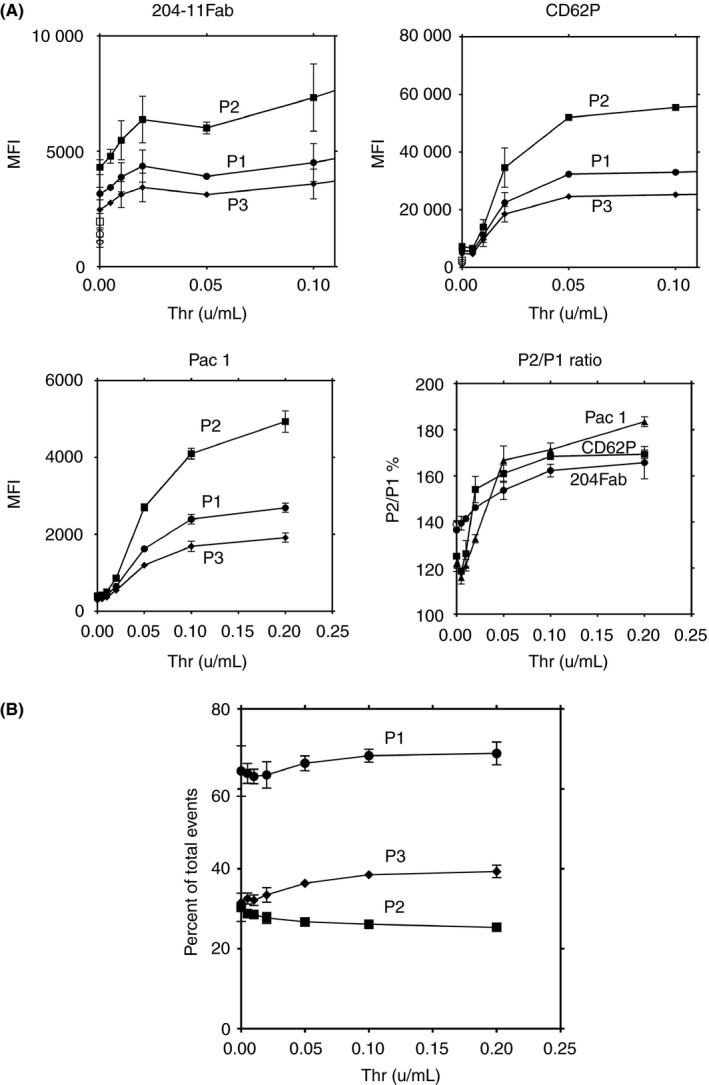
Concentration‐dependent receptor expression of activation‐dependent antigens P‐selectin and activated αIIbβ3. All data shown in this figure are representative of 3 experiments, giving similar results, using platelets from different donors. A, Washed platelets were activated by increasing thrombin concentrations and expressions of GPVI dimer, P‐selectin, and activated αIIbβ3 were measured using antibodies 204‐11 Fab, AK6, and PAC‐1, respectively. Expressions of receptors in whole platelets (P1), larger platelets (P2), and smaller platelets (P3) were measured. There are preferential and higher expressions of P‐selectin and activated αIIbβ3 (PAC‐1 binding) in P2 platelets compared to those in P1 and P3. B, Distribution of platelets to the large and small platelet populations. In the same experiments as panel A, the number of platelets in P1‐P3 are calculated as a percentage of events from the flow cytometry data. All the data were determined twice by flow cytometer, and their mean values were plotted with standard deviation

Thrombin‐induced changes in expressions of GPVI dimer, P‐selectin, and activated αIIbβ3 in P1‐P3 were measured using 204‐11 Fab, anti‐CD62P, and PAC‐1 antibodies, respectively. All 3 receptors increased dose dependently with thrombin, but increases in P2 were much greater than those in P1 and P3 (Figure 4A). Increase of GPVI dimer expression in P3 platelets was much less, and expressions of CD62P and active αIIbβ3 in P2 were more than 2‐fold those in P3. Since anti‐CD62P and PAC‐1 bind only to activated platelets, these results indicate that the major contributor to the measured increase in activation marker expression in the total population of activated platelets is the P2 population. Increases of receptor expression observed at 0.005‐0.01 units/mL thrombin were consistent with the thrombin sensitivity of a typical platelet aggregation assay.

Figure 4B shows the number of platelets distributed to P2 and P3 in terms of percentage of total events. If smaller platelets become larger upon activation (eg, platelet dimerization), the number in P2 should increase, while those in P3 should decrease. However, the percentage of total events increased slightly in P3 and decreased slightly in P2, suggesting that the platelets originally distributed in P2 would have increased their surface receptor expression. The slight decrease in the percentage of total events in P2 would be due to formation of aggregates.

### 3.4 Analysis of GPIb and GPVI shedding

**Figure 5:**
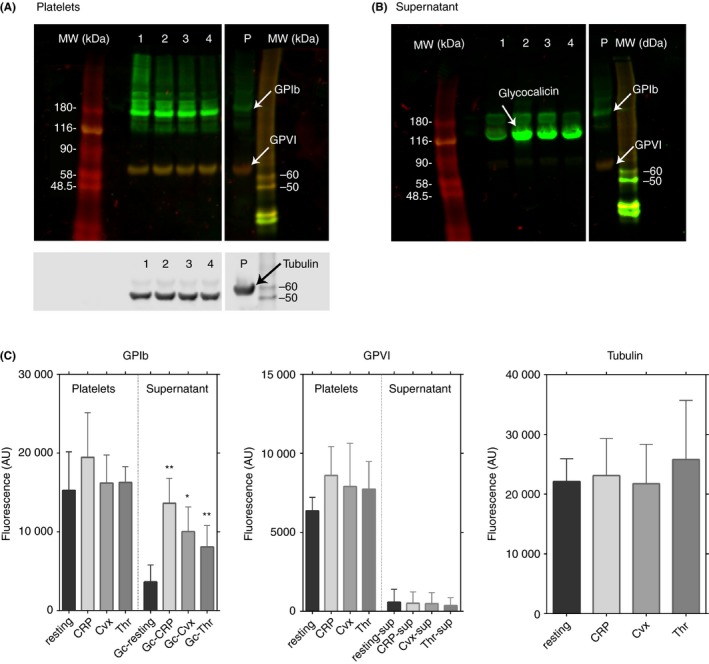
Immunoblotting analyses to determine if glycoprotein (GP) Ib and GPVI shedding occurs. Washed platelets were activated under the same conditions as used for the receptor expression analysis by flow cytometry and separated into the supernatant and pellet (platelets) fractions by centrifugation. Pellets are dissolved by SDS/urea so that they were the same volume as the supernatant samples and analyzed by immunoblotting. A, The immunoblotting of platelet pellets and (B) shows the results from the supernatants. Lane 1 (resting platelets), Lane 2 (crosslinked collagen‐related peptide [CRP‐XL]–activated platelets), Lane 3 (convulxin [Cvx]‐activated platelets), Lane 4 (thrombin‐activated platelets) P ( control platelets). C, The results from quantitation of these bands. Two SDS/immunoblotting analysis were done for the experiment and the graph indicates the means of band densities with standard deviation. *P ≤ .05, **P ≤ .005. These data indicate the shedding of GPIb from the activated platelets, but GPVI is not shed

Platelet activation by CRP‐XL, Cvx, or thrombin decreased surface GPIb expression, and activation by Cvx strongly decreased surface GPVI (Figures 2A‐D and 3A). Two mechanisms may cause these decreases—shedding or internalization. Shedding was assessed by activating washed platelets with CRP‐XL, Cvx, or thrombin under the same conditions as those used for flow cytometry; platelet pellet and supernatant were isolated and analyzed by western blotting. Western blotting with anti‐GPIb (Figure 5A‐C) detected low‐molecular‐weight GPIb (glycocalicin) in the platelet supernatant; its amount increased after platelet activation by any agonist. The pellet‐fraction lane contained one‐third the amount of loaded protein compared to the supernatant lane, so most of the GPIb is still in the platelets. However, the supernatant of resting or activated platelets contained similar amounts of faint bands of GPVI, with the same mobility as native GPVI and no degraded (shed) GPVI.^16^

### 3.5 Internalization of GPIb and GPVI in activated platelets

**Figure 6:**
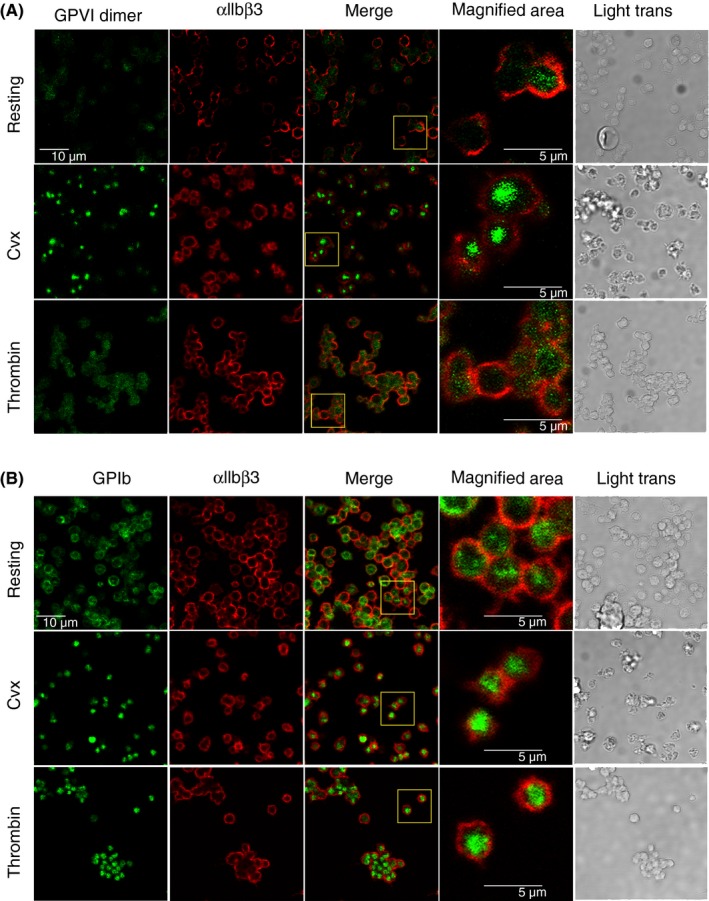
Analyses of internalization. Platelets are prelabeled with Alexa Fluor‐488–conjugated 204‐11 Fab (anti–GPVI dimer; A) or Alexa Fluor‐488–conjugated anti‐GPIb (clone 486805; B) and then activated with convulxin (Cvx) or thrombin. Platelets were fixed with formaldehyde/glutaraldehyde solution as described in the text and internalization of labeled antibody/antigen complex (green) was analyzed by confocal microscopy after staining with Alexa Fluor‐647–conjugated anti‐integrin αIIbβ3 (red) to allow clear visualization of the platelet plasma membrane. The data show that GPVI dimers of Cvx‐activated platelets and GPIb of Cvx‐ and thrombin‐activated platelets are internalized. The images are typical of the results from four experiments; in each experiment, 5‐8 fields of the sample were imaged for each condition

Platelets, prelabeled with Alexa Fluor‐488–conjugated antibodies against GPIb or GPVI, were activated by Cvx or thrombin, fixed, and immobilized on poly‐l‐Lys‐coated dishes. The immobilized platelets were stained with Alexa Fluor‐647–conjugated anti‐αIIbβ3 antibody to clearly visualize the cell membrane. Cvx‐activated platelets stained for GPVI show strong granule‐like fluorescence inside the platelets, while thrombin‐activated or resting platelets show only diffuse staining over the whole cell (Figure 6A). Cvx‐ and thrombin‐activated platelets stained for GPIb show similar strong staining inside the cells, and resting platelets show diffuse staining over the whole cell (Figure 6B). These results show that GPVI is internalized in Cvx‐activated platelets and GPIb is internalized in Cvx‐ and thrombin‐activated platelets.

**Figure 7:**
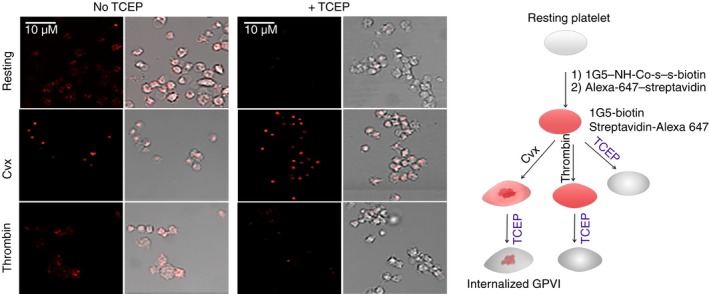
Analysis of glycoprotein (GP) VI internalization using TCEP (tris[2‐carboxyethyl]phosphine). Platelets were prelabeled with 1G5‐SS‐biotin and Alexa Fluor‐647–streptavidin and activated with convulxin (Cvx; 1 µg/mL) or thrombin (0.2 unit/mL). The platelets were reacted with 28 mmol/L TCEP in the presence of 5 mmol/L ethylenediaminetetraacetic acid for 10 min following with washing with citrate‐buffered saline. The platelets were resuspended in modified HEPES (4‐[2‐hydroxyethyl]‐1piperazineethanesulfonic acid)–Tyrode’s buffer, fixed with 1% paraformaldehyde, washed, plated on poly‐Lys coated MatTek dishes, and analyzed with confocal microscopy. TCEP removes all the fluorescence in the resting or thrombin‐treated platelets, indicating no internalization (ie, fluorescence confined to the platelet surface). However, the fluorescence of the Cvx‐treated platelets remains intact, confirming internalization. The right‐hand panel shows a schematic representation of how this experiment. This experiment was performed twice, with each giving similar results; in each experiment, 5‐8 fields of the sample were imaged for each condition

GPVI internalization was confirmed by treating thrombin‐ or Cvx‐activated 1G5‐SS‐biotin‐Alexa Fluor 647–streptavidin labeled platelets with the membrane‐impermeable reducing agent TCEP (Figure 7). The MFI values of these platelets changed after TCEP treatment from 693.5 to 357.7 (resting platelets), 999.4 to 1042.4 (Cvx‐activated platelets) and 671.1 to 448.2 (thrombin‐activated platelets). Figure 7 shows that TCEP removes most of the fluorescence in the resting or thrombin‐treated platelets, indicating no internalization (ie, fluorescence confined to the platelet surface), but the fluorescence of the Cvx‐treated platelets remains intact, confirming internalization.

### 3.6 Exposure of intracellular receptors on platelet spreading

**Figure 8:**
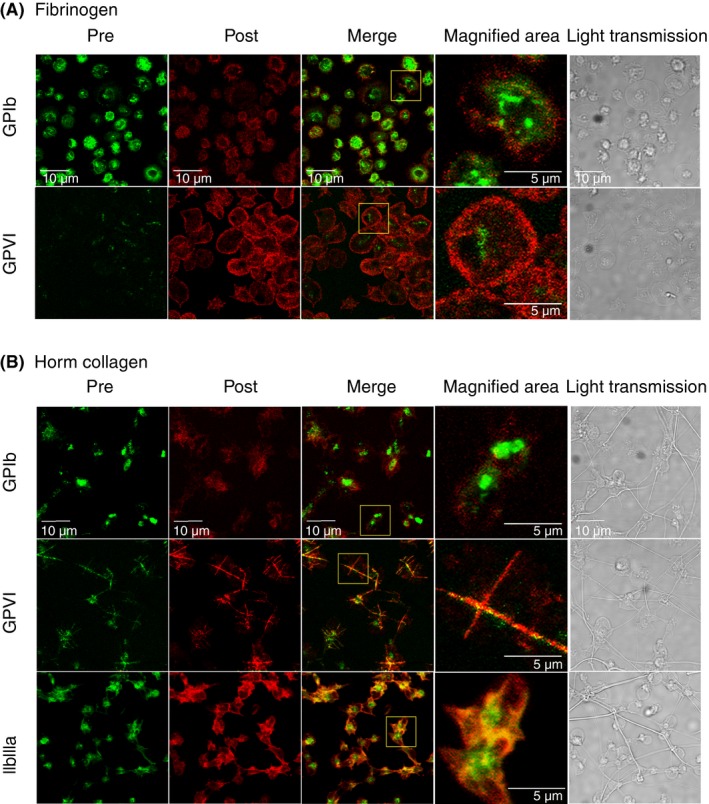
Colocalization analysis of prelabeled and postlabeled receptors on adhered platelets. Platelets were prelabeled with Alexa Fluor‐488–conjugated antibodies and were allowed to adhere on fibrinogen‐ or collagen‐coated surfaces. The adhered platelets were fixed and stained with Alexa Fluor‐647conjugates of the corresponding antibodies. For platelets adhered to immobilized fibrinogen, post‐staining shows the new expression of GPVI and GPIb, derived from intracellular GPVI and GPIb, on the surface membrane of spread cells, while the prestained receptors localized to the cell body. On the other hand, both pre‐ and post‐stained GPVI is essentially all confined to the clusters of GPVI adhered to the collagen fibers. This figure gives representative results from 1 of 3 experiments, all giving similar results; in each experiment, 5‐8 fields of the sample were imaged for each condition

Our flow cytometry data suggest that additional receptors are newly exposed on the platelet surface after platelet activation. Preexisting surface receptors were differentiated from newly exposed ones by allowing platelets labeled with Alexa Fluor‐488–conjugated receptor‐specific antibody to adhere and spread on immobilized collagen or fibrinogen and then post‐staining with the same antibody conjugated with Alexa Fluor‐647. For GPVI dimers, platelets were prestained with Alexa Fluor‐488 204‐11 Fab and post‐stained with AlexaFluor‐647‐1G5, since 204‐11 does not react with fixed platelets. Anti–GPVI dimer prelabeled platelets spread well on immobilized fibrinogen; prelabeled GPVI is localized on the cell body, but post‐stained GPVI appears in the membrane, at the edges of the lamellipodia, of spread cells (Figure 8A). The GPIb‐prestained cells are less spread but show differential staining similar to GPVI.

Platelets adhered to collagen (Figure 8B) have GPVI clusters along the collagen fibers, as previously reported,^15^ with prestained and post‐stained GPVI colocalized. This suggests that both preexisting and newly exposed GPVI moved to collagen’s platelet‐binding sites. Prestained GPIb is concentrated on the cell body, whereas post‐stained GPIb is diffuse and mostly localized to the lamellipodia of the spread platelet; this is different from the platelets adhered to fibrinogen, as collagen strongly activated the platelets. However, prestained and post‐stained αIIbβ3 are mainly colocalized, but strong prestaining is observed in the cell body. These results show that when platelets spread and a receptor does not interact with substrate, preexisting surface receptors reside at the cell body, while newly exposed receptors present at the edges of the lamellipodia in the spread platelet. If receptors react with substrate, preexisting and newly exposed receptors move to the binding site. Such conditions are typically present for GPVI on platelets adhering on fibrinogen and collagen.

## 4 DISCUSSION

This study demonstrates that platelet activation changes the levels of major glycoprotein receptors, either decreasing or increasing their surface expressions depending on both the receptor and the agonist. PRP, washed platelets, and fixed platelets (Figures 2 and 3A) all showed increased expression of surface αIIbβ2 upon activation by CRP‐XL, Cvx, or thrombin, but surface GPIb is decreased. Total and dimeric GPVI expressions markedly decreased after Cvx‐induced activation, but the other agonists induced similar or increased expression. 1G5 can activate platelets, so total GPVI expression in CRP‐XL‐ or thrombin‐induced determined with this antibody did not differ from the resting level. These results suggest that platelet activation increases surface expression of some receptors, like the integrins, but decreases expressions of GPIb and GPVI through other mechanisms.

GPIb and GPVI are susceptible to cleavage by metalloproteinases ADAM17 and ADAM10, respectively, after platelet activation, so their soluble forms, glycocalicin and 55‐KD GPVI, respectively, may be released into the medium.^16^, ^17^ Under the conditions of our flow cytometry assays, GPIb, as glycocalicin, was detectable in the supernatant, although most of it remained on the platelets (Figure 5), but almost no soluble GPVI was in the supernatant. No exogenous Ca2+ was added to our flow cytometry samples, so shedding of either glycoprotein should be minimized in our assay condition. Cvx‐activated platelets have strong patch‐like staining inside the cell, consistent with internalization of prestained GPVI (Figure 6). This was confirmed by our results showing that only the fluorescence of Cvx‐stimulated 1G5‐SS‐biotin‐streptavidin prelabeled platelets was resistant to cleavage by TCEP, whereas that of thrombin‐activated or resting platelets was not (Figure 7). Cvx‐ or thrombin‐activated GPIb‐prestained platelets showed similar internal fluorescent aggregates. Such GPIb internalization was previously observed,^18^, ^19^, ^20^, ^21^ but this is the first report of Cvx‐specific GPVI internalization. Platelets produce microparticles containing plasma membrane after activation,^22^ which could reduce the receptor level in the remaining plasma membrane, but expressions of other receptors increased under the same conditions, so this would contribute little to decreases in GPIb and GPVI.

Increased receptor expression after activation could be explained by the merging of OCS and granule membranes with the surface membrane, which translocate intracellular receptors to the platelet surface.^1^, ^2^, ^3^, ^4^, ^18^, ^19^ Thus, we can reasonably hypothesize that GPIb and GPVI expressions would first increase upon platelet activation and then decrease due to internalization, shedding, or both. Thrombin‐induced increase in surface GPVI and its Cvx‐induced decrease occur on similar time scales (Figure 3B), so GPVI internalization by Cvx must be rapid if intracellular GPVI is first cell‐surface–expressed and then internalized. The observed differential expressions of surface GPVI induced by thrombin and Cvx raises several possibilities about the internalization process: GPVI expression may be increased by thrombin activation through a mechanism similar to that for the increase in αIIbβ3, while direct binding of Cvx to GPVI may induce GPVI internalization. Our results suggest that large‐cluster formation may be required to induce strong internalization since CRP‐XL produces little internalization. Cvx is a tetramer of heterodimers,^23^ and while CRP‐XL is a crosslinked molecule, it is composed of single triple‐helical peptides, which do not bind to GPVI.^10^ Thus, only Cvx would form dense GPVI clusters large enough to induce internalization.

In contrast to GPVI, many agonists have been reported to decrease GPIb expression,^21^, ^24^ suggesting platelet activation in general would cause GPIb internalization. Electron micrographs suggested cytoskeletal involvement in GPIb internalization.^19^, ^20^, ^21^ However, αIIbβ3 binds to the cytoskeleton as well and is clustered after activation but does not internalize, so the precise mechanism of internalization remains inconclusive.

Using prestaining and post‐staining with receptor‐specific antibodies labeled with fluorophores that emit fluorescence of different wavelengths, we differentiated preexisting surface receptors in resting platelets from intracellular receptors newly exposed upon platelet activation. Prestained GPVI on fibrinogen‐adhered platelets localizes to the cell body in the center of the spread cell, while post‐stained GPVI localizes at the edges of the spread cell (Figure 8A). However, platelets adhered on collagen show colocalization of prestained and post‐stained GPVI clustered along the collagen fibers, as previously reported.^15^ These results indicate that the flat, spread membrane of fibrinogen‐adhered platelets would come from an intracellular resource, with newly exposed GPVI dispersed freely over the lamellipodia, but originally existing surface GPVI would remain at the same place. However, because of GPVI’s high affinity for collagen, prestained (green) and post‐stained GPVI (red) would move to the region of the membrane in contact with collagen fibers and become colocalized as membrane clusters on the fibers (yellow; Figure 8B). Distribution of prestained and post‐stained GPIb on platelets adhered to immobilized fibrinogen and collagen are essentially like that of GPVI on platelets adhered on fibrinogen, but since these platelets are not fully spread like the GPVI‐stained platelets, many of the spread cells show only a small separation of post‐ and prestaining. These results suggest that intracellular receptors become exposed in the spread membrane (lamellipodia) of adhered platelets and localize separately from the originally expressed surface receptors when they do not interact with substrate.

The P2/P1 ratio is increased upon activation, consistent with the larger platelets expressing more receptors on their surface compared to the smaller platelets. These results suggest that larger platelets are more activated than smaller platelets, as reported before.^25^, ^26^, ^27^, ^28^ Upon thrombin activation, P‐selectin and PAC‐1 expressions in the larger platelets are increased much more than in the total and smaller platelets (Figure 4A). At higher thrombin concentrations, P‐selectin and PAC‐1 expressions in P2 became about 2‐fold and 3‐fold higher than that of P1 and P3, respectively. Since gating by flow cytometry cannot totally separate the larger platelets from the smaller platelets, these results suggest that activation involving integrin activation and secretion would mainly occur in the larger platelet population.

The large‐platelet fraction isolated by differential centrifugation or flow cytometry was reported to be more active,^25^, ^26^, ^27^ and a relationship between activity and platelet size was reported.^28^ Although young platelets were thought to be large and more active, platelet size and age were indicated to independently affect platelet function.^29^, ^30^ Heterogeneity of platelet size was suggested to come from the heterogeneity of megakaryocytes^31^ and activation of the mechanistic target of rapamycin complex1 in megakaryocytes may contribute to elevated platelet volume,^9^ suggesting that megakaryocytes would produce different sizes of platelets and that the larger platelet subpopulation would have higher activities. Our data indicate that the larger subpopulation of activated platelets showed a higher level of agonist‐induced receptor expression compared to the total platelet population, supporting the previous reports describing that larger platelets have higher activities. In this context, it is very interesting that a larger MPV has been indicated to be a risk factor for cardiovascular disease in both men and women^7^, ^8^, ^32^ or just in men.^33^ Our results importantly suggest that the large‐platelet subpopulation would be the main contributor to higher platelet activity and our method, analyzing the receptor expression of resting and activated platelets by flow cytometry, would be a good method to analyze the risk factor of having larger platelets.

## RELATIONSHIP DISCLOSURE

RWF reports grants from the British Heart Foundation during the conduct of the study and personal fees from CambCol Ltd, outside the submitted work. SMJ reports grants from the British Heart Foundation during the conduct of the study. Dr Moroi reports nothing to disclose.

## AUTHOR CONTRIBUTIONS

MM designed and performed experiments, analyzed data, made figures, and wrote the manuscript. RWF critically read the manuscript and discussed the results with the other co‐authors. SMJ performed the confocal imaging, designed experiments, analyzed data, made figures, and wrote the manuscript.
